# MYCT1 alters the glycogen shunt by regulating selective translation of RACK1-mediated enzymes

**DOI:** 10.1016/j.isci.2022.103955

**Published:** 2022-02-22

**Authors:** Dong-Xue Ding, Yue Wang, Wei Yan, Wei-Neng Fu

**Affiliations:** 1 Department of Medical Genetics, China Medical University, Shenyang, China; 2 The Lundquist Institute for Biomedical Innovation at Harbor-UCLA Medical Center, Torrance, CA, USA

**Keywords:** Physiology, Animal physiology, Molecular biology

## Abstract

MYCT1 has been shown to function as a tumor suppressor in various tumors, but its role in metabolism has never been reported. Here, we showed that global inactivation of Myct1 in mice led to progressive accumulation of glycogen in the liver, which was accompanied by aberrant changes in intermediates of the glycogen metabolic pathway. Mechanistically, MYCT1 appeared to promote translation efficiency of PGM1, UGP2 and GSK3A in hepatic cells in a RACK1-dependent manner. Consequently, upregulation of the three enzymes enhanced the glycogen shunt. Our data reveal a critical role of MYCT1 as a switch for the glycogen shunt in tumor cells.

## Highlights

• *Myct1* depletion causes glycogen accumulation in mouse liver
• MYCT1 affects glycogen shunt in tumor and normal cells
• MYCT1 regulates translation efficiency of glycogen enzymes
• MYCT1 alters the glycogen shunt in a RACK1 dependent manner

## Graphical Abstract

**Figure:**
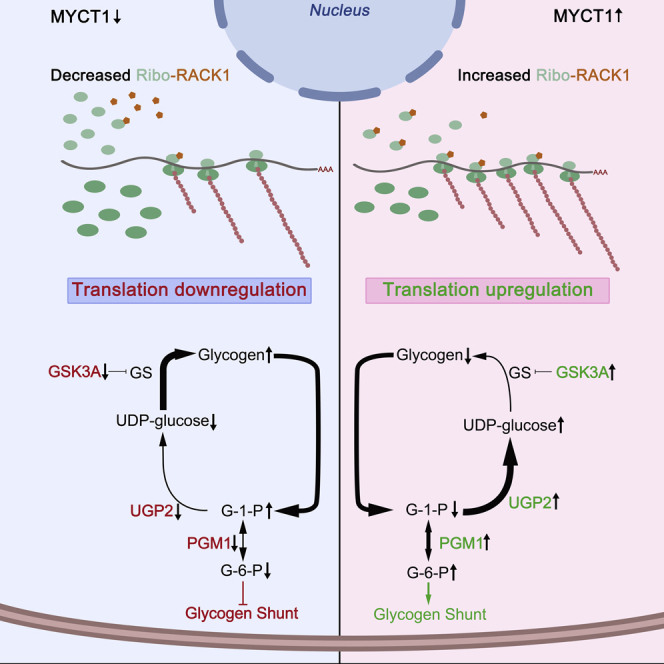

## Introduction

Glycogen metabolism plays a crucial role in maintaining glucose and energy metabolism homeostasis (Adeva-Andany et al., 2016). Recent advances in metabolic reprogramming suggest that glycogen metabolism may exert important biological effects through multiple metabolic pathways beyond its role as a reservoir of glucose including glycolysis and pentose phosphate pathways (PPP): the glycogen shunt, which is described as a condition when glucose is shunted to glycogen and subsequently consumed through glycolysis and other biosynthesis pathways even under the condition of adequate glucose, plays a critical role in tumorigenesis and development (Everts et al., 2014; Thwe et al., 2017; Shulman and Rothman, 2017; Dauer and Lengyel, 2019; Curtis et al., 2019a, 2019b).

Rothman et al. proposed that the metabolic flexibility of cancer cells involved four metabolic states including the respiration state of oxidizing non-glucose substrates under low glucose and normal oxygen, the glycolysis state with high glucose and reduced oxygen levels in tumors, and two states of the glycogen shunt allowing the cells to survive between the rapid transition in glucose and oxygen (Rothman and Shulman, 2021). Nutrition and hormonal stimuli instigate signaling cascades to activate transcription and translation factors involved in the regulation of metabolic flux. Previous studies have addressed the importance of allosteric regulation of glycogen synthase and glycogen phosphorylase, which can be activated or inactivated by GSK3 or PP1 (Beurel et al., 2015) and hormone stimuli signaling pathways (Hatting et al., 2018). It has been reported that the hypoxic microenvironment in tumor cells induced the HIF1α-dependent transcriptional upregulation of glycogen synthesis enzymes, such as phosphoglucomutase1 (PGM1), glucose-1-phosphate uridylyltransferase (UGP2), glycogen synthase 1 (GYS1) and 1,4-α glucan branching (GBE1), leading to the accumulation of glycogen (Mole et al., 2009; Pelletier et al., 2012; Pescador et al., 2010; Shen et al., 2010). Favaro et al. discovered that hypoxia-induced accumulation of glycogen in tumor cells was followed by a later increase of glycogen phosphorylase (PYGL). The glycogen degradation by PYGL was shunted to the pentose phosphate pathway which generated ribose 5-phosphate and nicotinamide adenine dinucleotide phosphate (NADPH), which was essential in protecting cells from reactive oxygen species (ROS) and strongly supported tumor growth (Favaro et al., 2012). However, when large glucose and oxygen changes happen, there may be insufficient time for transcriptional regulation for related gene expression to adapt.

MYCT1, a direct c-Myc target gene, regulates numerous downstream genes, thus leading to alterations in proliferation, apoptosis, migration, and invasion in various tumors (Yue et al., 2020; Xu et al., 2020). To further explore the functions of MYCT1, we generated a global *Myct1* knockout (KO) mouse line. Unexpectedly, the *Myct1* KO mice displayed progressive liver glycogen accumulation instead of tumor-related phenotypes, and this finding was consistent with the iTRAQ proteomics data obtained from our tumor research, showing enrichment of a series of glycogen synthesis enzymes (data not shown). In this study, we identified MYCT1 as a selective translational regulator of glycogen metabolizing enzymes through interactions with RACK1, a translation factor located on the 40S subunit ribosome (Nielsen et al., 2017). The translational upregulation of PGM1, UGP2 and GSK3A mediated by MYCT1 and RACK1 adjusted the flux of glycogen synthesis and glycogen shunt, which is crucial for modulating the tumor metabolic reprogramming.

## Results

### *Myct1* depletion causes abnormal glycogen accumulation in mouse liver

**Figure 1:**
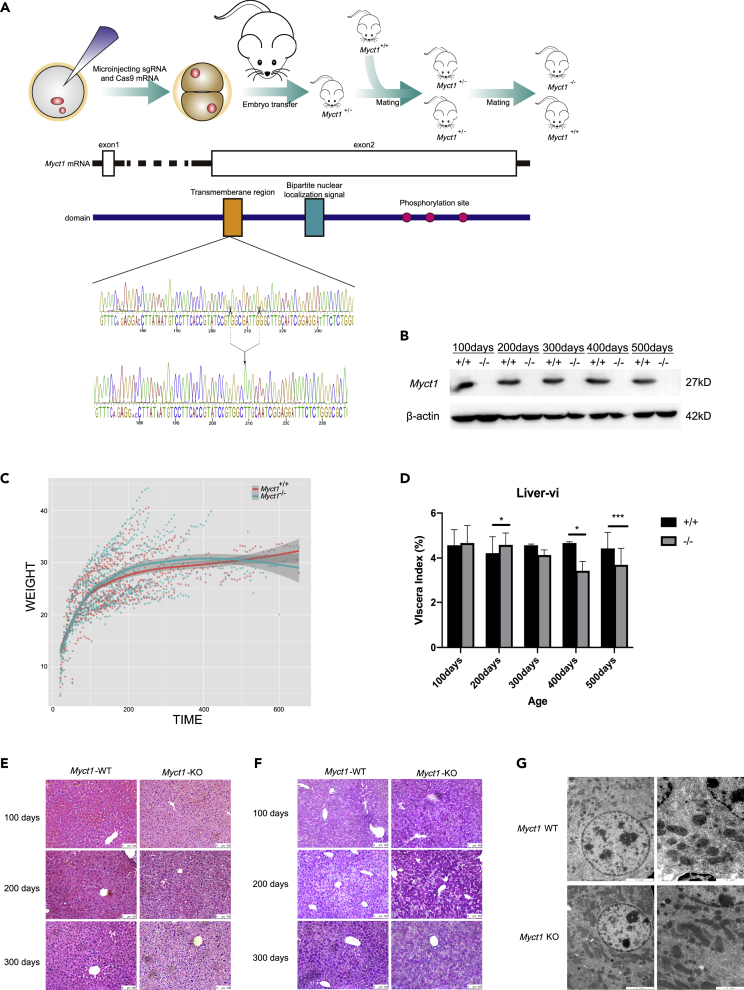
Characterization of the physiology and pathology characterization of the *Myct1*KO mouse model (A) Schematic representation of the strategy used to generate *Myct1* KO mice and identification of *Myct1* KO mice by Sanger sequencing. (B) Western blotting of Myct1 in the livers of WT and *Myct1* knockout mice at 100 to 500-day-old. (C) Growth curve for WT and *Myct1* knockout mice. WT: n = 38; *Myct1* KO: n = 38. (D) Liver viscera Index for WT and *Myct1* knockout mice at 100–500 days. WT: n = 28; *Myct1* KO: n = 28. (E) Histopathology analyses by HE staining of paraffin liver sections from 100- to 300-day-old littermates. Scale bar: 100 μm. (F) Histological analysis of the localization of glycogen by PAS staining of paraffin liver sections from 100- to 300-day-old littermates. Scale bar: 100 μm. (G) Transmission electron microscopic analyses of the localization of glycogen in liver samples from 200-day-old mice. Scale bar: 2 μm. The statistical analyses comparing variables of interest between the WT and KO groups were performed in R using a paired t test. The data are represented as the means ± SEMs from independent duplicates. ∗p < 0.05, ∗∗p < 0.01, ∗∗∗p < 0.001 and ∗∗∗∗p < 0.0001.

Using the CRISPR/Cas9 technology, we generated global *Myct1* KO mice. Sanger sequencing of the targeted region revealed that the KO allele contained an 8-nt deletion in the second exon which caused a frameshift in the coding region of *Myct1* mRNAs (Figure 1A). Using Western blotting, the antibodies raised against the C-terminus of MYCT1 detected robust expression of MYCT1 in wild-type, but not in KO liver lysates, suggesting that the mutant allele of *Myct1* that we generated was functionally null (Figure 1B).

To monitor the development of the KO mice, the body weights of 38 groups of *Myct1* KO mice and their WT littermates were measured weekly. As shown in the growth curves (Figure 1C), the bodyweight of the *Myct1* KO mice was higher than that of the WT mice before day 500, whereas the bodyweight of the *Myct1* KO mice decreased compared to that of the WT mice after day 500. With the calculation of the visceral index, hepatomegaly and liver atrophy were found at days 200 and 400, respectively (Figure 1D). We then performed hematoxylin-eosin (HE) staining to examine the pathological changes in the liver. The results showed that the *Myct1* KO hepatocytes displayed aberrant morphology, ranging from swelling, lighter cytoplasmic staining, and necrosis, which is similar to the phenotype of glycogenic liver disease (Figure 1E). The pathology appeared to get more severe with aging. Both periodic acid-Schiff (PAS) staining and transmission electron microscopy showed excessive glycogen storage, characterized by a large number of abnormal glycogen granules accumulated in the cytoplasm that cut off the endoplasmic reticulum (Figures 1F and 1G). To confirm the effect of *Myct1* on glycogen metabolism, which is also an important energy reserve of the brain, HE and PAS staining was conducted in brain tissues (Figures S1A and S1B). Neurons and astrocytes in the brain showed different degrees of apoptosis and necrosis by TUNEL staining (Figure S1E).

### MYCT1 Modifies Glycogen Metabolic Intermediates and Glycogen Shunt

**Figure 2:**
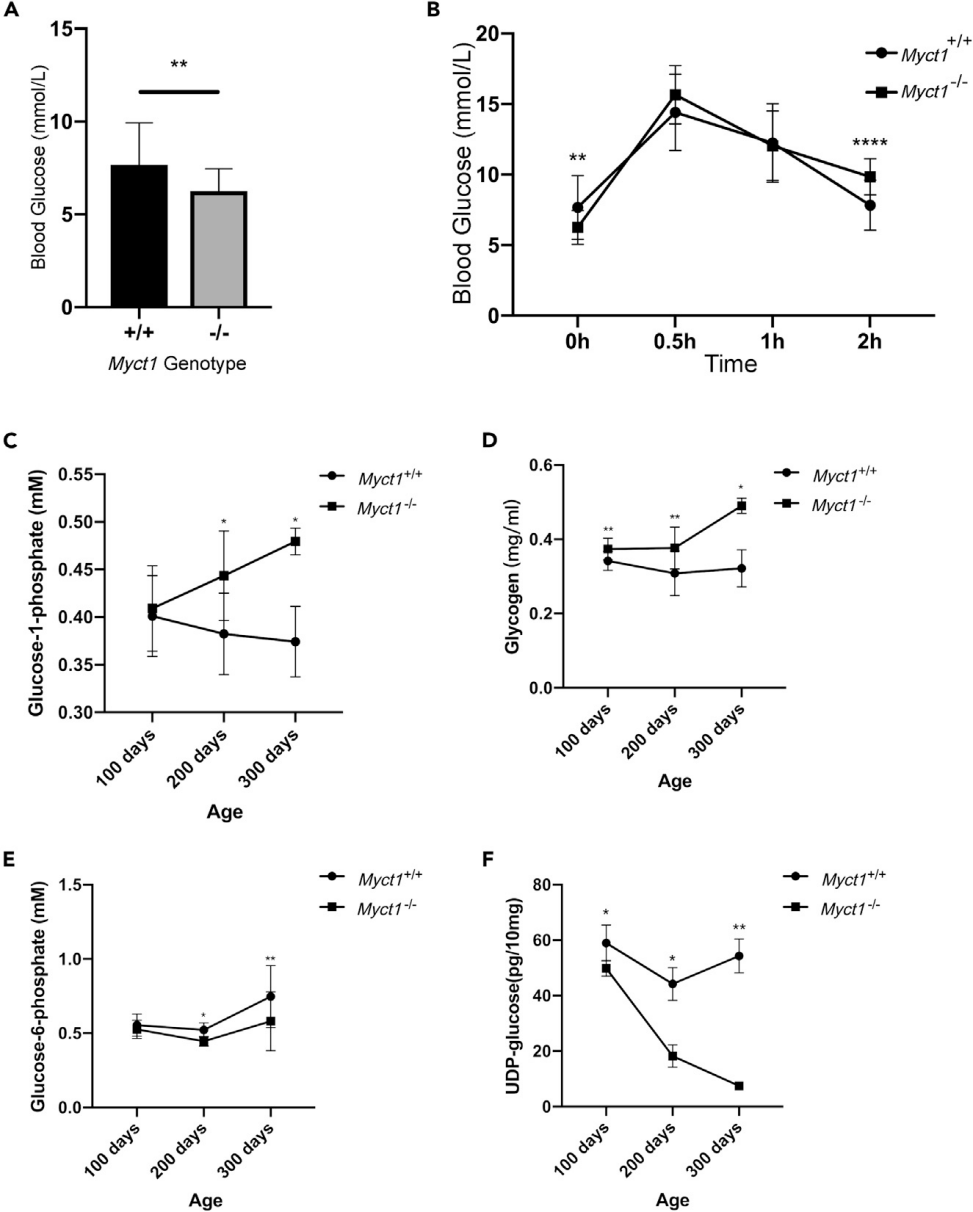
MYCT1 modifies glycogen metabolic intermediates and glycogen shunt (A) Fasting blood glucose levels of WT and *Myct1* KO mice. WT mice: n = 20; Myct1 KO mice: n = 20. (B) Intraperitoneal glucose tolerance test results of WT *Myct1* KO mice. WT mice: n = 20*Myct1* KO mice: n = 20. (C) Glucose-1-phosphate levels in liver tissues (10 mg) from WT and *Myct1* KO mice measured by colorimetric quantitation. WT mice: n = 10; *Myct1* KO mice: n = 10. (D) Glycogen levels in liver tissues (10 mg) from WT and *Myct1* KO mice measured by colorimetric quantitation. WT mice: n = 10; *Myct1* KO mice: n = 10. (E) Glucose-6-phosphate levels in liver tissues (10 mg) from WT and *Myct1* KO mice measured by colorimetric quantitation. WT mice: n = 10; *Myct1* KO mice: n = 10. (F) The UDP-glucose levels in liver tissues (10 mg) from WT and *Myct1* KO mice measured by the UDPG-ELISA kit. WT mice: n = 10; *Myct1* KO mice: n = 10. The statistical analyses comparing variables of interest between the WT and KO groups were performed in R using a paired t test. The data are represented as the means ± SEMs from independent duplicates. ∗p < 0.05, ∗∗p < 0.01, ∗∗∗p < 0.001 and ∗∗∗∗p < 0.0001.

To assess the impact of *Myct1* ablation on glycogen metabolism in the liver, the fasting blood glucose levels were measured (FBG) and intraperitoneal glucose tolerance tests (IPGTT) were performed. Compared with WT mice, *Myct1* KO mice had significantly lower FBG and higher 2-h IPGTT glucose levels, suggesting an imbalanced transformation between glycogen and glucose with an identical onset of pathological impairment (Figures 2A and 2B). Glycogen and its intermediates were also determined, including glucose-6-phosphate (G6P), glucose-1-phosphate (G1P) and uridine diphosphate glucose (UDPG), in the livers *Myct1* KO and WT littermates. The gradual deterioration of phenotypes was accompanied by comparable increases in G1P and glycogen and a sharp decrease in UDPG and G6P in the liver of *Myct* KO mice compared to those of WT mice (Figures 2C–2F). The deletion of *Myct1* directed more carbon sources to glycogen stored in cells and reduced the diversion to glycolysis through G6P, which is quite similar to the opposite pattern of glycogen shunt in tumor cells. Taken together, these observations point to a critical role of MYCT1 in the regulation of glycogen shunt in the liver.

### MYCT1 Controls Glycogen Shunt in tumor and normal cells

Given the strong association between MYCT1 expression and glycogen metabolism, we selected three different types of cells, including HepG2 cells with low MYCT1 expression, Huh7 cells with high MYCT1 expression and LO2 cells, to represent three different metabolic conditions and both tumor and normal cells as well (Figure S2A). We transiently transfected MYCT1 vectors and siMYCT1 to the three types of cells and determined the levels of metabolites in MYCT1-overexpressing (MYCT1-OE) and MYCT1-knockdown (MYCT1-KD) cells.

**Figure 3:**
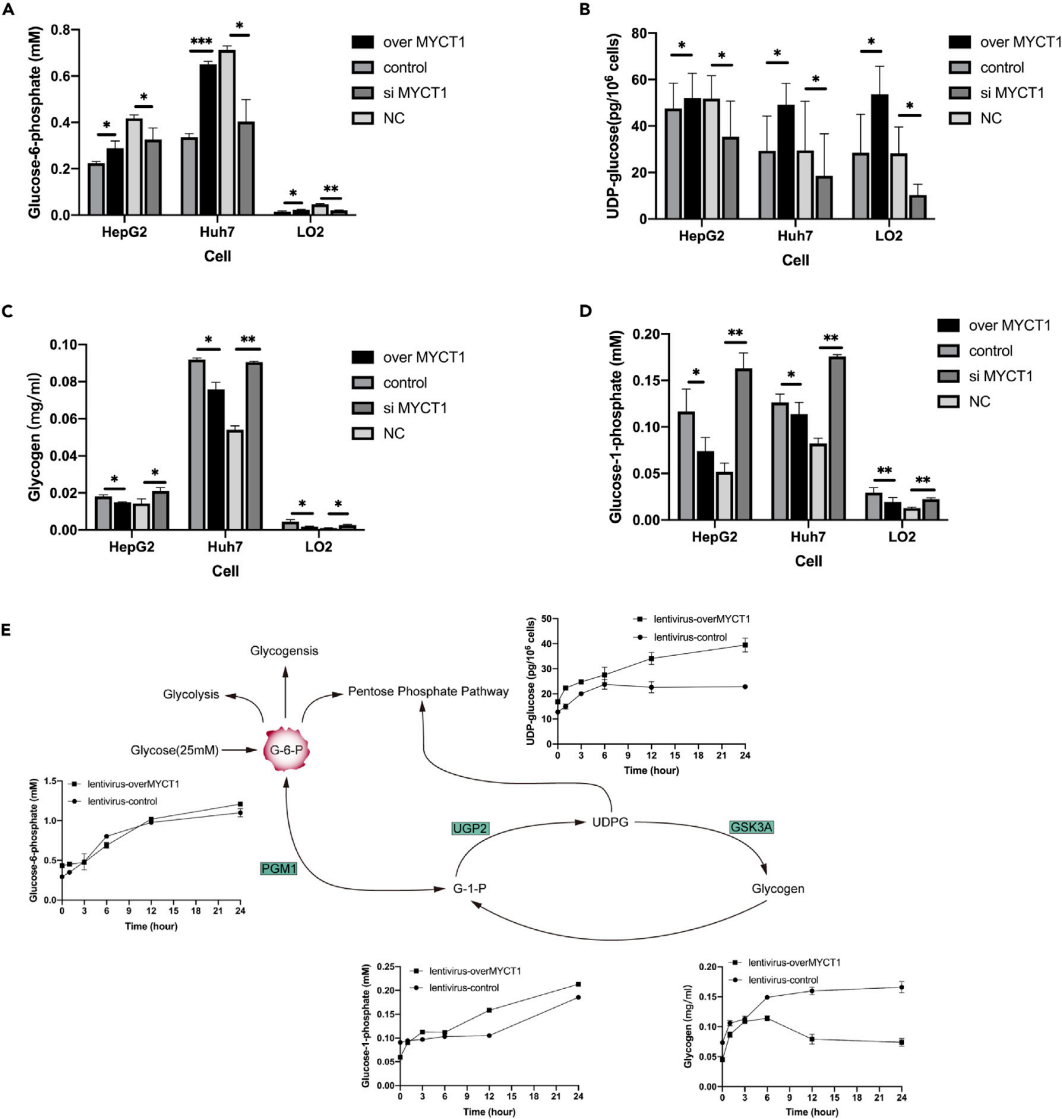
MYCT1 controls glycogen shunt in both tumor and normal cells For a Figure360 author presentation of this figure, see https://doi.org/10.1016/j.isci.2022.103955. (A) The glucose-1-phosphate levels in the overMYCT1/control vector and siMYCT1/NC transfected HepG2, Huh7 and LO2 cells measured by colorimetric quantitation. n = 3. (B) The glucose-6-phosphate levels in the overMYCT1/control vector and siMYCT1/NC transfected HepG2, Huh7 and LO2 cells measured by colorimetric quantitation. (C) The glycogen levels in the overMYCT1/control vector and siMYCT1/NC transfected HepG2, Huh7 and LO2 cells measured by colorimetric quantitation. (D) The UDP-glucose levels were determined by the UDPG-ELISA kit. (E) Glucose-1-phosphate, UDP-glucose, glucose-6-phosphate and glycogen production curves under high glucose conditions in the lentivirus-overMYCT1/control HepG2 cells. The statistical analyses comparing variables of interest between the treatment and control groups (n = 3) were performed in R using paired t test. Data are expressed as means ± SEMs from independent duplicates. ∗p < 0.05; ∗∗p < 0.01; ∗∗∗p < 0.001.

MYCT1-OE groups showed elevated G6P and UDP-glucose contents and lower glycogen and G1P contents, which changed the opposite in MYCT1-KD groups (Figures 3A–3D). Similar to the pattern of glycogen shunt, the MYCT1 mediated effect on glycogen metabolism caused the elevated conversion from G1P to G6P, a central hub for glycolysis, and more UDP-glucose was discharged for the consumption of glycosylation and PPP. Notably, the metabolite quantities in control groups showed significant variations among three different cells, and higher levels were found in tumor cells, particularly in those with high MYCT1 expression. To further define whether hepatocytes could adjust the glycogen shunt in the presence of adequate glucose, we stimulated stable-MYCT1 HepG2 cells with a high glucose concentration of 25 mM and recorded the metabolites fluctuations (Figure 3E). At the first 6 h of stimuli, all intermediates and glycogen in the pathway increased simultaneously. As the storage of glycogen reached the plateau ∼6 h later, the levels of G6P and UDPG continuously increased at an accelerated rate, whereas the levels of glycogen began to decline along with the slower increase of G1P. We also assessed the glycolysis flux using the Seahorse Glycolysis Stress Test. The extracellular acidification rate was greatly enhanced in MYCT1 stable HepG2 cells (Figure S2B). These data implied that MYCT1 triggered the glycogen shunting to other pathways through G6P and UDPG, the two cross centers of glucose metabolic network under the high level of glucose (Rajas et al., 2019).

To validate the effect of MYCT1 in regulating the glycogen shunt, we analyzed mRNA levels *Myct1* in various tumors, including bladder cancer, breast cancer, esophageal cancer, glioma, head and neck cancer, hepatocellular carcinoma, lung adenocarcinoma, lung squamous cell carcinoma, ovarian cancer, gastric cancer, renal clear cell carcinoma and renal papillary cell carcinoma, based on the information provided in The Cancer Genome Atlas (TCGA) (Figure S2C), and our analyses showed that MYCT1 was highly expressed in renal clear cell carcinoma (Lai et al.,2021), which is a typical glycogen-rich carcinoma (Bannasch et al.,2017; Vranic et al., 2020), indicating the regulatory function of MYCT1 in glycogen shunt.

### MYCT1 Regulates the Translation Efficiencies of Glycogen Enzymes

To identify the mechanism underlying glycogen shunt in the *Myct1* KO liver, we screened the expression of several glycogen metabolizing enzymes, including PGM1, UGP2, GSK3A, GYS2, GBE1, PYGL and AGL using Western blotting (Figure S3A). Notable declines in PGM1, UGP2 and GSK3A levels were detected in the *Myct1* KO liver samples. The expression of PGM1, UGP2 and GSK3A was detected in three different types of cells transiently transfected by the MYCT1 vector and two different types of cells infected by lentivirus-MYCT1 (Figures S3B and S3C). The results showed that MYCT1 selectively increased the protein expression of PGM1, UGP2 and GSK3A. To eliminate the effect of the new protein produced by the deletion of eight bases of *MYCT1* on the expression levels of glycogen metabolizing enzymes, we designed the same 8-base deletion *MYCT1* overexpression plasmid and siMYCT1 as the knockout mice. The results showed that the mutated *MYCT1* did not affect the expression of PGM1, UGP2 and GSK3A in HepG2 cells (Figure S3D).

**Figure 4:**
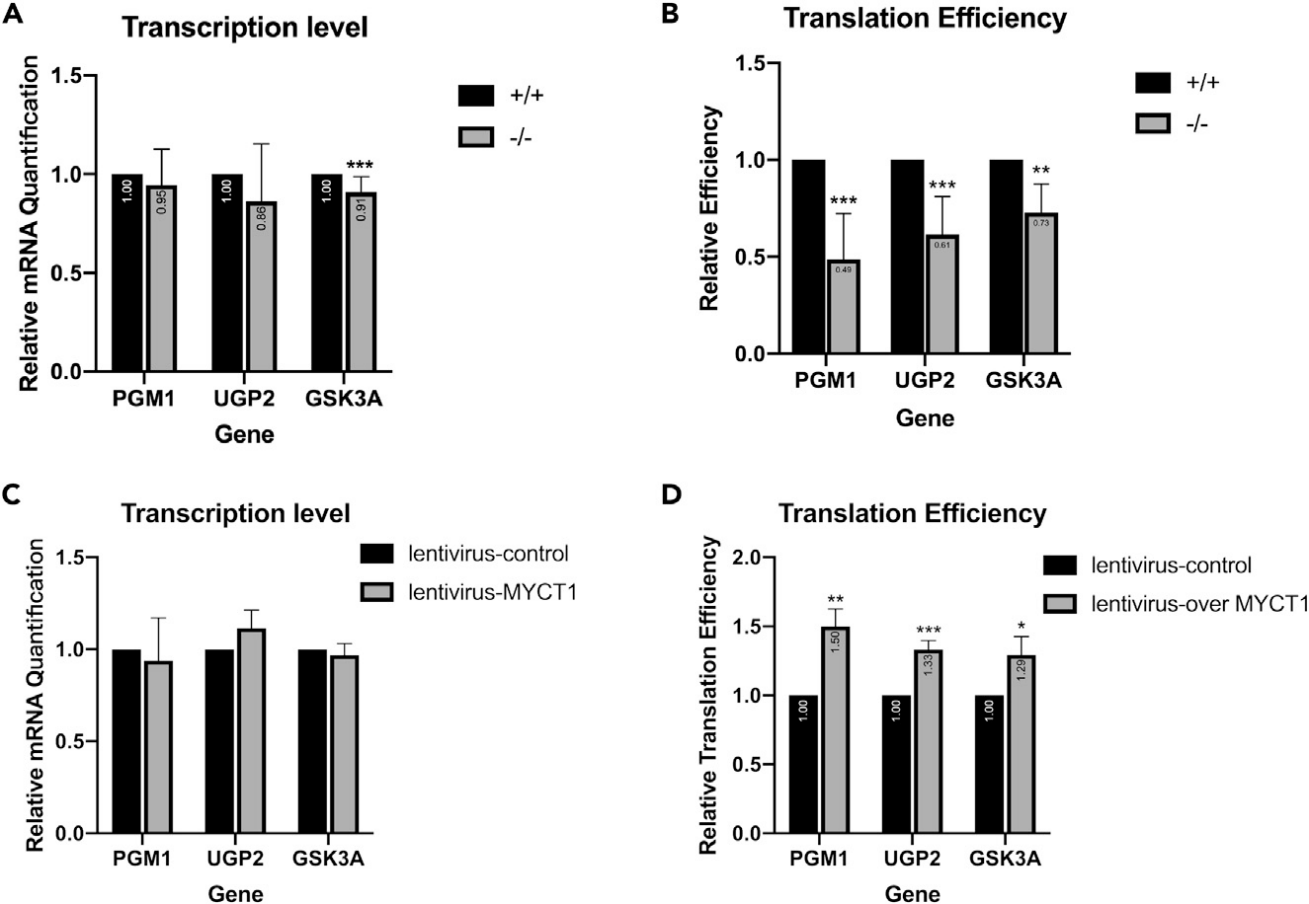
MYCT1 regulates the translation efficiencies of glycogen enzymes (A) qPCR analyses of mRNA levels of the three glycogen metabolizing enzymes (Pgm1, Ugp2 and Gsk3a) in liver tissues from WT and *Myct1* KO mice. WT mice: n = 15; *Myct1* KO mice: n = 15. (B) Relative TEs of the three glycogen-metabolizing enzymes *Pgm1*, *Ugp2* and *Gsk3a* in liver tissues from WT and *Myct1* KO mice. WT mice: n = 6; *Myct1* KO mice: n = 6. (C) Relative mRNA quantitation of glycogen metabolizing enzymes PGM1, UGP2 and GSK3A in lentivirus-overMYCT1/control HepG2 cells. n = 3. (D) Relative translation efficiencies of glycogen metabolizing enzymes PGM1, UGP2 and GSK3A in lentivirus-overMYCT1/control HepG2 cells. n = 3. The statistical analyses comparing variables of interest between the treatment and control groups were performed in R using paired t test. Data are expressed as means ± SEMs from independent duplicates. ∗p < 0.05; ∗∗p < 0.01; ∗∗∗p < 0.001.

After verifying the effect of MYCT1 on the protein level of metabolic enzymes, we next analyzed the relative abundance of their mRNAs and the translational activities of these mRNAs. Ribosome nascent-chain complex-bound mRNAs (RNC-mRNAs) were extracted by sucrose density ultracentrifugation, and the translation efficiency (TE) was calculated (Wang et al., 2013).*Myct1* KO liver tissues, the mRNA levels of *Pgm1*, *Ugp2* and *Gsk3a* did not show significant differences or were slightly decreased (less than 10%), compared to those found in the WT mice (Figure 4A). However, the TEs were markedly declined (Figure 4B). Myct1 depletion greatly reduced the TEs of *Pgm1*, *Ugp2* and *Gsk3a* to 49%, 61 and 73%, respectively, and these decreases showed a correlation with the corresponding protein levels. We then performed the same experiments in HepG2 cells infected by lentivirus-MYCT1. Stable MYCT1 overexpression did not influence transcription levels (Figure 4C) and upregulated translation of the above-mentioned three enzymes by 50%, 29 and 30%, respectively (Figure 4D). These data strongly suggested MYCT1 may regulate the expression of the genes involved in glycogen shunt at translational levels.

### MYCT1 interacts with RACK1 and Affected the Enrichment of RACK1 on ribosomes

**Figure 5:**
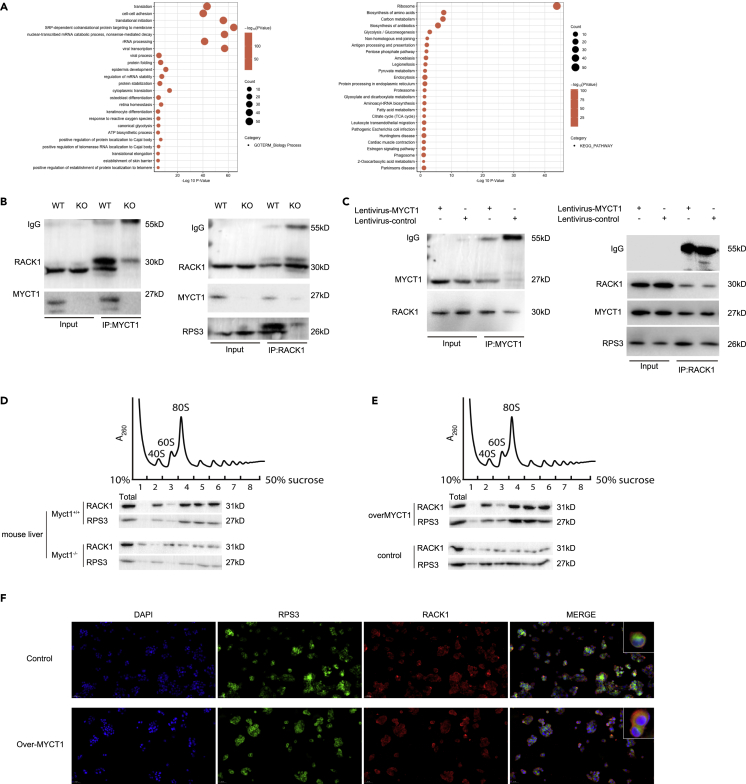
MYCT1 interacts with RACK1 and affected the enrichment of RACK1 on ribosomes (A) GO/KEGG analyses of MYCT1-targeted immunoprecipitation followed by mass spectrometry in mouse liver tissues. (B) Co-immunoprecipitation of MYCT1 and RACK1 in WT/*Myct1* KO mice liver tissues. (C) Co-immunoprecipitation of MYCT1 and RACK1 in lentivirus-over MYCT1/control HepG2 cells. (D) Representative polysome profile (top) complemented with RACK1 from WT/*Myct1* KO mice liver tissues. The fractions were analyzed by immunoblotting. (E) Representative polysome profile (top) complemented with RACK1 from lentivirus-MYCT1/control HepG2 cells. The fractions were analyzed by immunoblotting. (F) Immunofluorescence stain of RACK1 and RPS3 in lentivirus-overMYCT1/control HepG2 cells. Scale bar: 50 μm.

To explore the specific mechanism of MYCT1 in translational control, MYCT1-targeted co-immunoprecipitation followed by mass spectrometry was conducted in mouse liver tissues and GO/KEGG analyses revealed one large set of MYCT1 interactors centered on translation and translation initiation regulation (Figure 5A). Among those translation factors associated with ribosomes, RACK1 drew our interest because it is known to interact with most of the ribosomal proteins and to regulate the translation of metabolic enzymes in many model organisms (Nielsen et al., 2017). We first confirmed RACK1 and MYCT1 as interacting partners by co-immunoprecipitation in both liver and HepG2 cells (Figures S4A and S4B), and immunofluorescence of MYCT1 and RACK1 in WT mouse liver and HepG2 cells (Figures S4C and S4D). Surprisingly, when we used RPS3, a conserved RACK1-interacting protein on ribosomes (Gallo et al.,2018), as a positive control for RACK1-targeted co-immunoprecipitation, clear differences in the binding of RPS3 and RACK1 between WT/Myct1 KO mouse liver samples were detected (Figure 5B) and MYCT1-lentiviral/control HepG2 cells (Figure 5C). Specifically, ribosomal protein RPS3 bonded with RACK1 was decreased and increased in *Myct1* KO mouse liver and stable-MYCT1 HepG2 cells, respectively. These results suggest that MYCT1 might affect the enrichment of RACK1 on ribosomes and thus, the translation capacity as previously reported (Gallo et al., 2018). To explore the effects of MYCT1 on the enrichment of RACK1 on ribosomes, we performed polysome analysis followed by Western blotting. The amounts of RACK1 associated with monosomes and polysomes showed marked differences between WT to *Myct1* KO mice (Figure 5D), and RACK1 was more enriched on monosomes and polysomes in HepG2 cells infected lentivirus-MYCT1 than in controls (Figure 5E). Consistently, immunofluorescence staining of RACK1 and RPS3 showed that MYCT1 enhanced the binding of RACK1 to ribosomes (Figure 5F). These discoveries suggest that MYCT1 is essential for the ribosome binding of RACK1.

### MYCT1 alters the glycogen shunt in a RACK1 dependent manner

**Figure 6:**
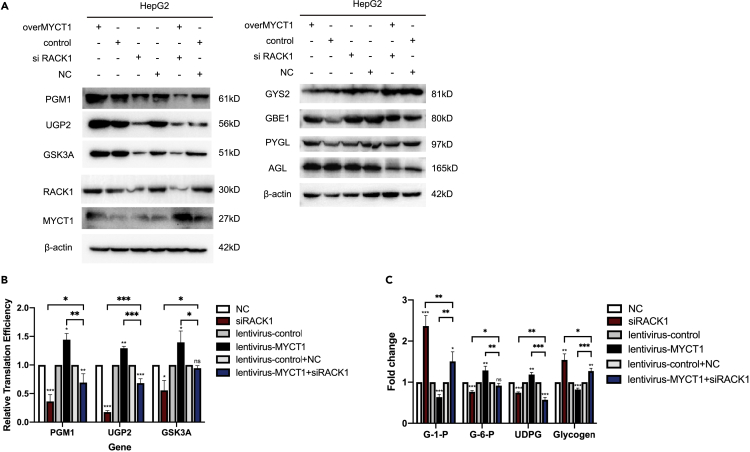
MYCT1 alters the glycogen shunt in a RACK1 dependent manner (A) Relative protein levels of glycogen metabolizing enzymes in lentivirus-MYCT1/control HepG2, lentivirus-MYCT1+siRACK1/control + NC and siRACK1/NC HepG2 cells. (B) Relative TEs of PGM1, UGP2 and GSK3A in NC/siRACK1, lentivirus-control/lentivirus-overMYCT1, lentivirus-control + NC/lentivirus-overMYCT1+siRACK1 HepG2 cells. (C) Glucose-1-phosphate, glucose-6-phosphate, UDPG and glycogen levels of NC/siRACK1, lentivirus-control/lentivirus-overMYCT1, lentivirus-control + NC/lentivirus-overMYCT1+siRACK1 HepG2 cells measured by colorimetric quantitation and ELISA. The statistical analyses comparing variables of interest between the treatment and control groups (n = 3) were performed in R using paired t test. Data are expressed as means ± SEMs from independent duplicates. ∗p < 0.05; ∗∗p < 0.01; ∗∗∗p < 0.001.

RACK1 is a ribosomal protein that controls the translation of specific mRNA, which also serves as the interface between translational and regulatory networks. To investigate the functional interplay between MYCT1 and RACK1, we measured the protein expression of glycogen enzymes in siRACK1 transfected stable-MYCT1 HepG2 cells with RACK1 knockdown and MYCT1 overexpression, which served as controls, by Western blotting. As expected, MYCT1 unregulated PGM1, UGP2 and GSK3A while the co-transfected cells showed decreased expression of the three enzymes (Figure 6A). In parallel with the protein levels, the decreased expression of RACK1 reduced the TEs and the co-transfection group showed no increase at the translation level, which indicated that the knockdown of RACK1 attenuates the translation upregulation induced by MYCT1 (Figure 6B). Consistent with the enzyme alternations, abundant levels of G1P and glycogen accumulated in cells after RACK1 knockdown, whereas sharp decreases in G6P and UDPG were detected (Figure 6C). The effects of MYCT1 on the glycogen shunt were also blocked by siRACK1. Taken together, these data suggest that RACK1 regulates the expression of glycogen metabolizing enzymes and the glycogen shunt at translational levels; more importantly, the MYCT1-mediated adjustment of glycogen metabolism was achieved via the RACK1-mediated regulation of translation.

## Discussion

The glycogen shunt, as first described in muscle (Shulman and Rothman, 2001) and cerebral (Shulman and Rothman, 2015), is known to be critical for the survival of normal and neoplastic cells during large shifts in nutrition. However, how cells adjust the glycogen shunt to support their demands for growth and development remains elusive. Our data unveil a regulatory mechanism of the glycogen shunt in which MYCT1 upregulates the expression of multiple enzymes at the translational level and redistributes glucose from glycogen to other metabolic pathways.

The synthesis and utilization of glycogen require the coordinated action of a series of enzymes. The deficiency of any of these enzymes could induce glycogen synthesis and utilization dysfunctions. PGM1 is designated a glycogen storage disease type XIV and is characterized by a failure to utilize glycogen due to inhibition of the formation of glucose 6-phosphate from glucose 1-phosphate (Marquardt et al.,2014). The metabolic profile of UGP2 mutant cells showed reduced abilities to produce UDP-glucose and synthesize glycogen and impaired protein glycosylation (Zeng et al., 2019; Perenthaler et al., 2020; Wolfe et al., 2021). GSK3A acts as a negative regulator in the hormonal control of glycogen synthesis by phosphorylating and inhibiting GS activity, and hence glycogen synthesis through glycogen deposition in *Gsk3a* KO mouse livers (Sowton et al.,2020). In our *Myct1* KO mouse model, we identified three enzymes affected by the ablation of *Myct1* and found excessive liver glycogen storage, which was consistent with a known deletion phenotype, suggesting a change in the glycogen shunt.

Our most striking finding was that MYCT1 can reprogram the homeostasis of glycogen shunt by changing the expressions of three enzymes simultaneously. The majority of prior studies on the glycogen shunt have mainly focused on the effects of a single enzyme on glycogen synthesis or utilization, including glycogen synthase (GSase), glycogen phosphatase (GPase) (Favaro et al., 2012), fructose bisphosphatase (FBase) and pyruvate kinase (PK). The study on yeast conducted by Shulman and Rothman showed that a futile cycle involving FBase allowed the flow of glucose to progress from G6P to fructose 1,6-bisphosphate (FBP) and back, which reduced the input flux of the glycogen shunt. Other researchers have shown that PK played a similar role in both yeast and cancer cells in the glycogen shunt and acted as a flux switch between glycogen and pyruvate (Mazurek, 2011; Israelsen et al., 2013; Yang and Lu, 2013). In the present study, the MYCT1-mediated adjustment of glycogen shunt was based on the coordinated regulation of multiple enzymes rather than being confined to the activation of one specific enzyme. The upregulation of GSK3A enhanced the inhibition of glycogen synthase and reduced glycogen synthesis. The higher expression of PGM1 caused the elevated conversion from G1P to G6P, a central hub for glycolysis, the PPP, *de novo* lipogenesis and the hexosamine pathway. The collaborative increases of UGP2 and GSK3A discharged more UDP-glucose for the consumption of glycosylation and PPP. These changes mediated by MYCT1 promoted the utilization of glycogen by cells through the shunt to other pathways.

Modulation of the TEs exhibits a profound correlation with the reprogramming of biological processes. Known mechanisms involve mTOR-mediated translation initiation regulation (Saxton and Sabatini, 2017; Thoreen, 2017) and mRNA stability (Huang et al., 2018; Wang et al., 2015). Unlike the mTOR signaling pathway, which promotes general protein synthesis, RACK1, as a scaffold ribosomal protein interacting with translation factors, RNA binding proteins and signal transduction molecules, can selectively control the TE of specific mRNAs. Our study discovered the binding of MYCT1 and RACK1 and demonstrated that the distinct translation control of PGM1, UGP2 and GSK3A depended on both the expression and ribosome enrichment of RACK1. These observations offer a valuable perspective on the regulation of translation in glycogen metabolism.

Post-transcription regulation is a rapid and efficient system to control the expression of pre-existing RNA to response to the dramatic change of nutrition and energy. Translation control of specific genes mediated by MYCT1/RACK1 provides a precise means to fine-tune glycogen shunt assisting tumor cells to achieve metabolic homeostasis.

### Limitations of the study

Our findings were based on the mouse model *in vivo* and tumor cells *in vitro*. Although we verified the correlation between MYCT1 expression level and glycogen shunt in the TCGA database, its significance for disease prognosis analysis and treatment in tumors characterized by glycogen metabolism needs to be verified in abundant clinical data.

## STAR★Methods

### Key resources table

**Table:** 

REAGENT or RESOURCE	SOURCE	IDENTIFIER
**Antibodies**		

Rabbit polyclonal anti-MYCT1	Abcam	Cat#ab139945; RRID:AB_2861408
Rabbit polyclonal anti-PGM1	ProteinTech	Cat# 15161-1-AP; RRID:AB_2161415
Rabbit polyclonal anti-UGP2	ProteinTech	Cat# 10391-1-AP; RRID:AB_2272775
Rabbit polyclonal anti-GSK3A	ProteinTech	Cat# 13419-1-AP; RRID:AB_2247995
Mouse monoclonal anti-Actin	ProteinTech	Cat# 66009-1-lg; RRID:AB_2687938
Rabbit polyclonal anti-GBE1	ProteinTech	Cat#20313-1-AP; RRID:AB_10697658
Rabbit polyclonal anti-PYGL	ProteinTech	Cat# 15851-1-AP; RRID:AB_2175014
Rabbit polyclonal anti-GYS2	ProteinTech	Cat# 22371-1-AP; RRID:AB_2879091
Rabbit polyclonal anti-AGL	ProteinTech	Cat# 10391-1-AP; RRID:AB_2272775
Goat polyclonal anti-RACK1	R&D System	Cat# AF3434; RRID:AB_2111955
Normal rabbit IgG	Cell Signaling Technology	Cat# 2729; RRID:AB_1031062
Rabbit IgG HRP linked	GE Healthcare	Cat#NA931; RRID: AB_772210
Alexa FluorTM 488 Phalloidin	Thermo Fisher Scientific	Cat#A12379
donkey anti-goat IgG-TR	Santa Crus	Cat#SC-2783; RRID:AB_641160
Mouse IgG HRP linked	GE Healthcare	Cat#NA931; RRID: AB_772210
Rabbit polyclonal anti-RACK1	Cell Signaling Technology	Cat# 5432; RRID:AB_10705522
Rabbit polyclonal anti-RPS3	ProteinTech	Cat# 11990-1-AP; RRID:AB_2180758

**Bacterial and virus strains**		

DH5a for cloning	Thermo Fisher Scientifific	Cat#18265017

**Chemicals, peptides, and recombinant proteins**		

Cycloheximide	MCE	Cat#HY-12320
DTT	Solarbio	Cat#D8220; CAS : 3483-12-3
Puromycin	Gibco	Cat# A1113803; CAS: 58-58-2
ANTI-RNase (15-30 U/mL)	Invitrogen	Cat#AM2692
D-Glucose	Gibco	Cat#A2494001
TRIzol reagent	Invitrogen	Cat#15596026
Protease inhibitor cocktail	Bimake	Cat#B14001

**Critical commercial assays**		

Periodic Acid Schiff Stain Kit	Solarbio	Cat#G1281
Haematoxylin-Eosin Stain Kit	Solarbio	Cat#G1120
PrimeScript™ RT reagent Kit (Perfect Real Time)	Takara	Cat#RR036A
TaKaRa BCA Protein Assay Kit	Takara	Cat#T9300A
PrimeSTAR® HS (Premix)	Takara	Cat#R040Q
TB Green® Premix Ex Taq™ II (Tli RNaseH Plus)	Takara	Cat#RR820A
Glucose-1-Phosphate Colorimetric Assay Kit	Sigma-Aldrich	Cat# MAK098
Glucose-6-Phosphate Colorimetric Assay Kit	Sigma-Aldrich	Cat#MAK014
Glycogen Colorimetric Assay Kit	Sigma-Aldrich	Cat#MAK016
Human/mouse UDPG ELISA Kit	Runyu	N/A
Immunoprecipitation (IP/CoIP) Kit	Absin	Cat#abs955
Seahorse XF Glycolysis Stress Test Kit	Agilent	Cat#103020-100
PrimeScript™ 1st Strand cDNA Synthesis Kit	Takara	Cat# 6110A

**Deposited data**		

Raw and analyzed data	This paper Mendeley Data	https://doi.org/10.17632/53262nkr3j.1
Affinity-based mass spectrometry	This paper Mendeley Data	https://doi.org/10.17632/gtpc2k68km.1

**Experimental models: Cell lines**		

HepG2	Chinese Academy of Sciences	Cat#TCHu72; CSTR:19,375.09.3101HUMTCHu72
HuH7	Chinese Academy of Sciences	Cat#TCHu182 CSTR:19375.09.3101HUMTCHu182
LO2	Chinese Academy of Sciences	N/A

**Experimental models: Organisms/strains**		

Mouse: C57BL/6	Animal Experimental Center of China Medical University	N/A

**Oligonucleotides**		

MYCT1 siRNA GUUCUCCCAACGUAAGCCCAGC	This paper	N/A
Negative control UUCUCCGAACGUGUCACGUTT	This paper	N/A
RACK1 siRNA GACCAACTATGGAATTCCTT	This paper	N/A
Primers, see Table S1	This paper	N/A

Recombinant DNA		

pCDNA3.1-CMV-MYCT1(Human,NM_025107.3)-SV40promoter-neo	SyngenTech	N/A
pCDNA3.1-CMV-MYCT1(Human,NM_025107.3,c.232-239del)-SV40promoter-neo	SyngenTech	N/A
pCDNA3.1-CMV-MCS-SV40promoter-neo	SyngenTech	N/A
pLV-hef1a-nNeongreen-P2A-puro-WPRE-CMV-MYCT1	SyngenTech	N/A

Software and algorithms		

ImageJ	ImageJ	https://imagej.nih.gov/ij/
R studio	R studio	https://www.rstudio.com/about/
GraphPad prism v9.0	GraphPad	RRID:SCR_002798

### Resource availability

#### Lead contact

Further information and request for resources and reagents should be directed to and will be fulfilled by the lead contact, Wei-Neng Fu (wnfu@cmu.edu.cn).

#### Materials availability

This study did not generate new unique reagents.

#### Data and code availability

Immunoprecipitation mass spectrometry data and original data have been deposited at Mendeley and publicly available as of the date of publication. The DOI and accession numbers are listed in the key resources table. This paper does not report any original code. Any additional information required to reanalyze the data reported in this paper is available from the lead contact upon request.

### Experimental model and subject details

#### Animals

Experiments on mice were performed in the animal facility of China Medical University (Shenyang, China) with the approval of the Institutional Animal Care and Use Committee (IACUC) of China Medical University. Mice were maintained in the IVC Animal Experiment System under a light-dark cycle (12 h) and specific pathogen-free conditions with access to food and water. All procedures were approved by the China Medical University Laboratory Animal Welfare and Ethical Committee. C57BL/6J mice heterozygous for *Myct1* KO were provided by the Animal Experimental Center of China Medical University and homozygous *Myct1* KO/wild-type (WT) littermates of all ages were obtained by brother-sister mating. The genotypes were confirmed at the DNA/mRNA level by Sanger sequencing. Littermates of the same sex with different genotypes were randomly assigned to experimental groups. Both male and female mice at 100–500 days were involved in the measurement of body weights and visceral index. Mice of random sex at 100–300 days were conducted histopathology analyses and detection of intermediates, protein expression and mRNA quantitation.

#### Cell lines

HepG2, Huh7 and LO2 cells were purchased from Cell Bank, Type Culture Collection, Chinese Academy of Sciences. HepG2, Huh7 and LO2 cells were cultured in DMEM, MEM and RPMI1640 (HyClone) containing 10% FBS (Invitrogen), 2 mM L-glutamine, 100 U/mL penicillin and 100 mg/mL streptomycin at 37°C in a humidified incubator containing 5% CO2. All cells utilized were tested negative for Mycoplasma and identified by STR-based method for confirmation.

### Method details

#### Histochemistry

Tissues were fixed in 4% PFA and embedded in paraffin. Embedded tissues were cut into 4-μm sections. Histochemical analyses for HE and PAS staining were carried out manually by kit construction. All histochemical analyses were performed successfully on a minimum of 6 animals per group.

#### Transmission electron microscopy

Animals were perfused, and tissues were fixed with 2.5% glutaraldehyde and 2% paraformaldehyde in 0.1 M phosphate buffer. Tissue slices were post-fixed in 1% osmium tetroxide, stained with 0.8% potassium ferrocyanide, dehydrated, and embedded in EPON resin. Ultrathin sections collected on copper grids were stained with 2% uranyl acetate in water and lead citrate solution. Electron micrographs of livers were taken using a Tecnai G2 F20 (FEI) 200 kV FEG with a CCD Eagle 4k x 4k transmission electron microscope. All TEM analyses were successfully performed on samples from 3 animals per group, with a minimum of 15 pictures per sample analyzed by a blinded investigator.

#### Fasting glucose and glucose tolerance test

Fasting glucose and glucose tolerance tests were carried out following an overnight fast (12 h). Mice were administered 2 mg/g glucose by i.p. injection, and blood glucose was assayed from the tail vein using a glucometer (Contour Next, Bayer Healthcare) at 0.5, 1, 1.5 and 2 h.

#### Metabolite determination

Tissues and cells extracted for biochemical analysis were snap-frozen in liquid nitrogen and stored at −80°C until use. G1P, G6P and glycogen were measured spectrophotometrically using a commercial kit (Sigma-Aldrich, USA). UDPG was detected using a human/mouse UDP-glucose ELISA kit.

#### Seahorse XF glycolysis stress test

The Extracellular Acidification Rate (ECAR) was measured using a Glycolysis Stress Test Kit and eXF96 Extracellular Flux Analyzer (Seahorse Bioscience) according to the manufacturer’s protocol. In brief, 10,000 cells were plated in 100 μl of their standard growing media and cultured overnight. On the day of the measurement, cells were washed with XF media and incubated in a CO2-free incubator at 37°C for 2 h to establish equilibration before loading. ECAR measurements were taken before and after the addition of glucose (10 mM), oligomycin (1 mM) and 2-DG (50 mM) and used to calculate glycolysis, glycolytic capacity and glycolytic reserve.

#### Ribosome nascent-chain complex-bound mRNA (RNC-mRNA) extraction

RNC extraction was performed as described by Zhang et al. with certain modifications. In brief, cells and tissues were pre-treated with 100 μg/mL cycloheximide for 15 min followed by ice-cold phosphate-buffered saline washes and the addition of 2 ml RB buffer (20 mM pH 7.4 HEPES-KOH; 15 mM MgCl2; 200 mM KCl; 2 mM DTT; 4 U/mL RNase inhibitor; 1× protease cocktail inhibitor; 100 μg/mL cycloheximide; 1% Triton 100). After 30 min, cell lysates were scraped and transferred to ice-cold 1.5 mL tubes. Cell debris was removed by centrifugation at 16200 g for 10 min at 4°C. The RNA concentration of the supernatant was determined. Supernatants were transferred to the surface of 20 mL of 30% sucrose in RB buffer. RNCs were pelleted with ultracentrifugation at 150000 g for 5 h at 4°C. Total RNA and RNC-RNA were extracted by using TRIzol RNA extraction reagent (Invitrogen) following the manufacturer's instructions. Both total RNA and RNC-RNA were prepared from three independent experiments.

#### Quantitative real-time PCR

Reverse transcription was performed using PrimeScript™ RT Master Mix (Perfect Real Time) (Takara). Quantitative real-time PCR was performed using TB Green® Premix Ex Taq™ II (Tli RNaseH Plus) on an ABI 7500 (Applied Biosystems). The primer sequences for real-time PCR are described in Table S1.

#### Protein extraction and western blot

Tissues and cells were homogenized in lysis buffer containing 25 mM HEPES (pH 7.4), 150 mM NaCl and protease inhibitor cocktails (Bimake). After sonication on ice, the suspension was centrifuged at 12000 g for 30 min to remove cell debris. The protein concentration was determined by BCA (Taraka). 50 mg protein was resolved by SDS-PAGE and immunoblotted with antibodies against MYCT1 (1:1000, Abcam), PGM1 (1:1000, ProteinTech), UGP2 (1:1000, ProteinTech), GSK-3a (1:1000, ProteinTech), GYS2 (1:1000, ProteinTech), GBE1 (1:1000, ProteinTech), PYGL (1:1000, ProteinTech), AGL (1:1000, ProteinTech), RACK1 (1:1000, Cell Signaling), RPS3 (1:1000, ProteinTech), and β-actin (1:10000, ProteinTech). Washed membranes were incubated with species-appropriate HRP-linked secondary antibodies (1:4000), and visualization was performed using ECL reagent.

#### Polysome analysis

A total of 1 × 10ˆ7 cells and 10 mg tissues were pre-treated with 100 μg/mL cycloheximide for 15 min followed by ice-cold phosphate-buffered saline washes and the addition of 2 mL RB buffer (20 mM pH 7.4 HEPES-KOH; 15 mM MgCl2; 200 mM KCl; 2 mM DTT; 4 U/mL RNase inhibitor; 1x protease cocktail inhibitor; 100 μg/mL cycloheximide; 1% TritonX-100). After 30 min, cell lysates were scraped and transferred to ice-cold 1.5 ml tubes. Cell debris was removed by centrifugation at 16200 g for 10 min at 4°C. Lysates containing 200 mg of total RNA were run through 10%–50% sucrose gradients using a Beckmann Coulter SW41 Ti rotor at 150,000 g for 4°C for 5 h. Gradients were fractionated using a Biocomp piston gradient fractionator. Protein was TCA-precipitated from the fractions and methanol-precipitated to remove residual sucrose. Pellets were resuspended in loading buffer and boiled at 95°C for 5 min for Western blotting.

#### Immunoprecipitation

Immunoprecipitation (IP) was carried out according to the manufacturer’s instruction of the IP/CoIP kit (Absin). Both tissues and cells were lysed with IP buffer, sonicated, and centrifuged. The supernatants were precleaned with Protein A/G beads and subjected to IP by incubating them with antibodies overnight and Protein A/G beads for 3 h at 4°C. The beads were washed three times with wash buffer, and the protein complexes were denatured using SDS sample buffer for western blot analysis.

#### Immunofluorescence

Tissue paraffin-embedded sections were subjected to deparaffinization and rehydration protocols and boiled in 0.01 M sodium citrate buffer (pH 6) at 100°C for 10 min for antigen retrieval. The slides were removed from heat and allowed to stand at RT in the buffer for 20 min. Cells were grown on sterile glass coverslips and fixed in 4% paraformaldehyde in TBS (pH 7.4) for 15minat RT. The samples were washed twice with ice-cold PBS. Both tissue and cell sections were blocked with 5% serum or BSA for 2 h at RT. Slides were incubated with diluted primary antibody overnight at 4°C and conjugated secondary antibody for 2 h at RT. The sections were incubated with DAPI for 1 min and rinsed with TBS. The slides were finally mounted with a drop of mounting medium.

### Quantification and statistical analysis

The results are presented as the mean ± SEM of independent experiments. Unless otherwise indicated, significance between two variables was analyzed using a paired or Student’s t test, performed with R studio and GraphPad Prism software. The following p values were statistically significant: p value > 0.05 (∗), p value > 0.01 (∗∗), and p value > 0.001 (∗∗∗).

### Additional resources

Further information and requests for reagents may be directed to and will be fulfilled by the corresponding author Wei-Neng Fu (wnfu@cmu.edu.cn).

## Supplemental information

Document S1. Figures S1–S4 and Table S1

Figure360. An Author Presentation of Figure 3
