# Supplementary material for: MYCT1 alters the glycogen shunt by regulating selective translation of RACK1-mediated enzymes
Source: iScience. 2022 Feb 22;25(3):103955. doi: 10.1016/j.isci.2022.103955 (PMC8908216; doi:10.1016/j.isci.2022.103955)
Supplement: Document S1. Figures S1–S4 and Table S1 [file mmc1.pdf]

**iScience, Volume *25***

## Supplemental information

# MYCT1 alters the glycogen shunt by regulating selective translation of RACK1-mediated enzymes

**Dong-Xue Ding, Yue Wang, Wei Yan, and Wei-Neng Fu**

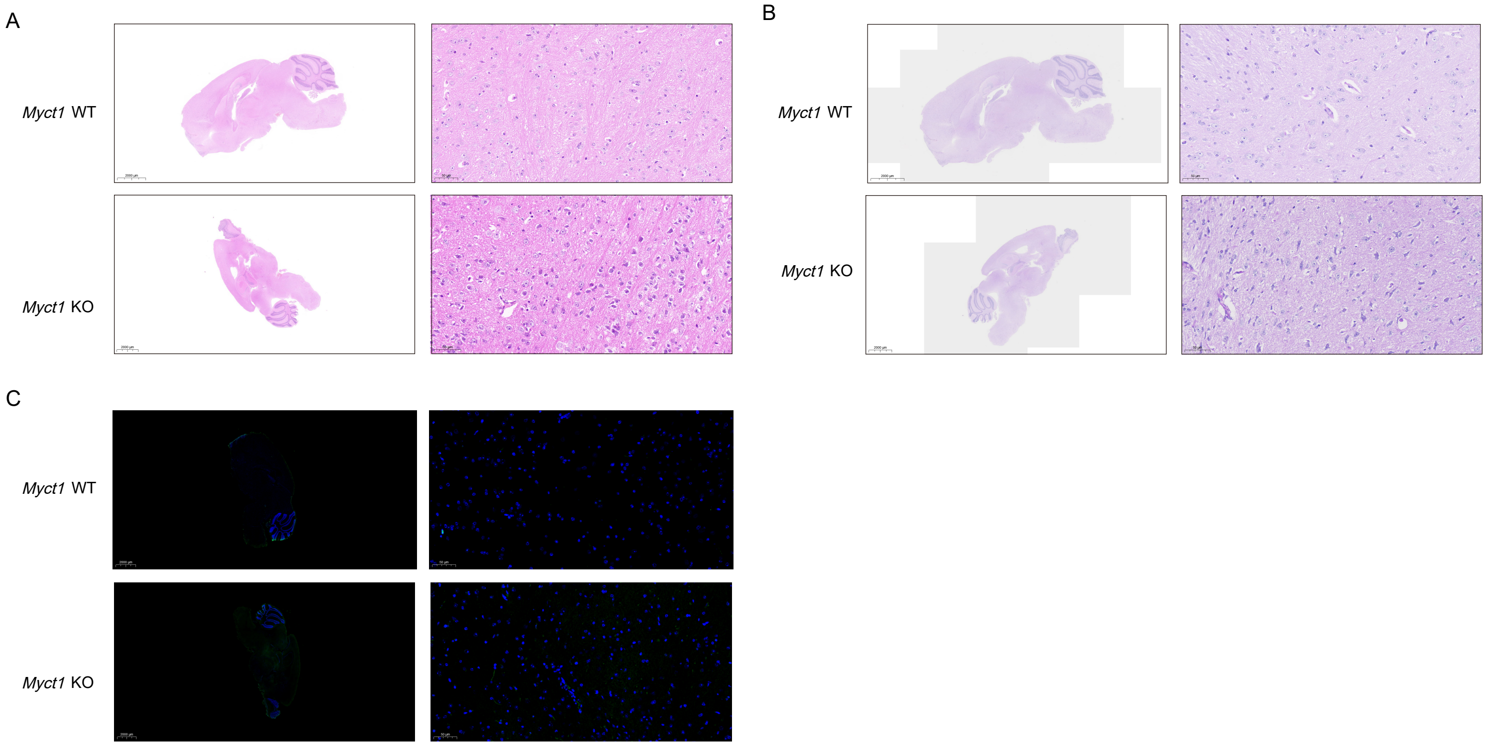


Figure S1. Characterization of the *Myct1* KO mouse model in brain. Related to Figure 1.

(A) Histopathology analysis by HE staining of paraffin brain sections from WT and *Myct1* KO littermates. Scale bar: 2000 μm and 50 μm.

(B) Histological analysis of the localization of glycogen by PAS staining of paraffin brain sections from WT and *Myct1* KO littermates. Scale bar: 2000 μm and 50 μm.

(C) TUNEL staining of paraffin brain sections from WT and *Myct1* KO littermates. Scale bar: 2000 μm and 50 μm.

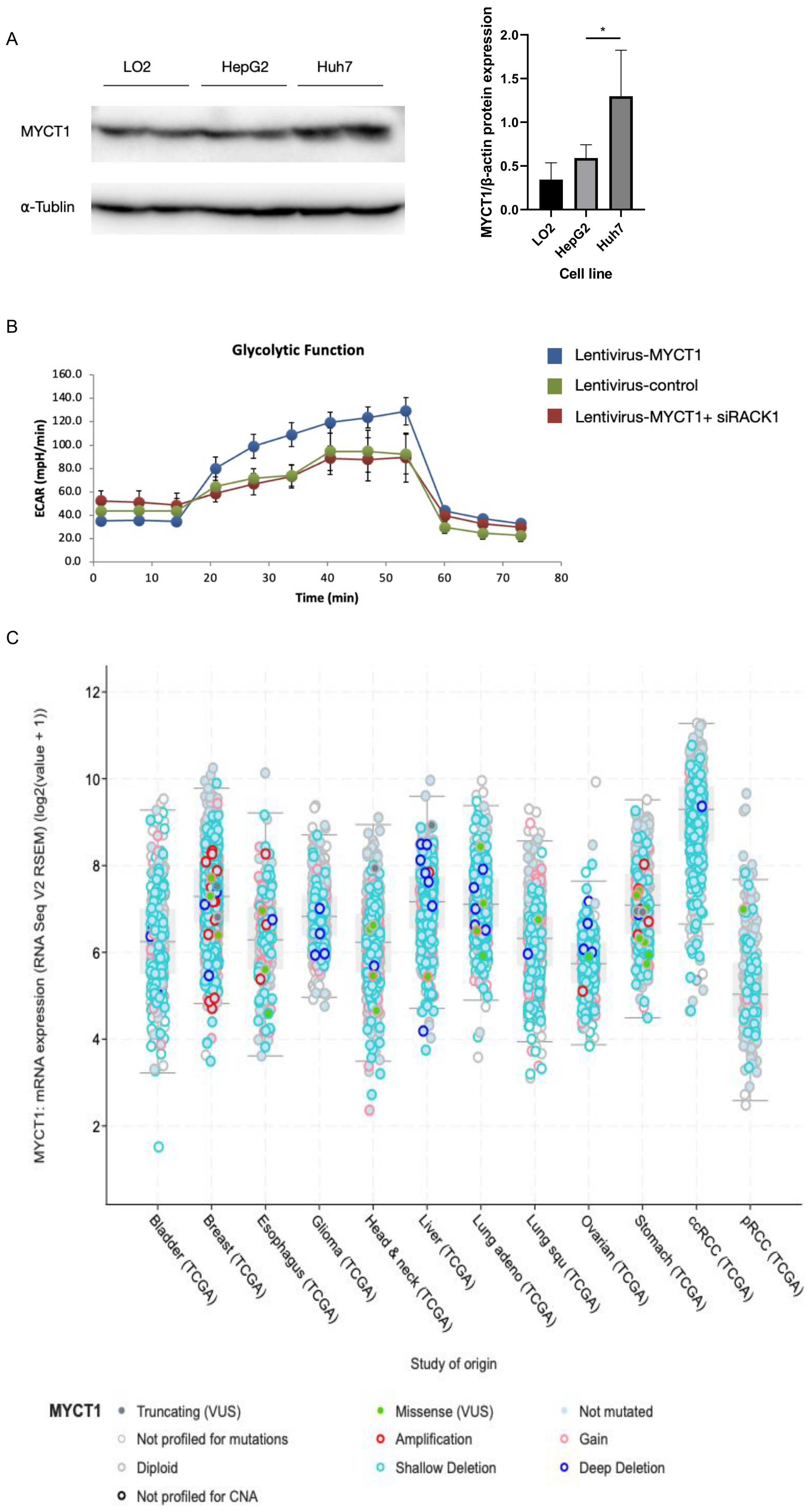


Figure S2. MYCT1 controls glycogen shunt in both tumor and normal cells. Related to Figure 3.

(A) Western blotting of MYCT1 in LO2, HepG2 and Huh7 cells.

(B) The glycolytic stress curves in lentivirus-overMYCT1/lentivirus-control/lentivirus-overMYCT1+siRACK1 HepG2 cells.

(C) Plots of MYCT1 mRNA expression from TCGA data in bladder cancer, breast cancer, esophageal cancer, glioma, head and neck cancer, hepatocellular carcinoma, lung adenocarcinoma, lung squamous cell carcinoma, ovarian cancer, gastric cancer, renal clear cell carcinoma and renal papillary cell carcinoma.

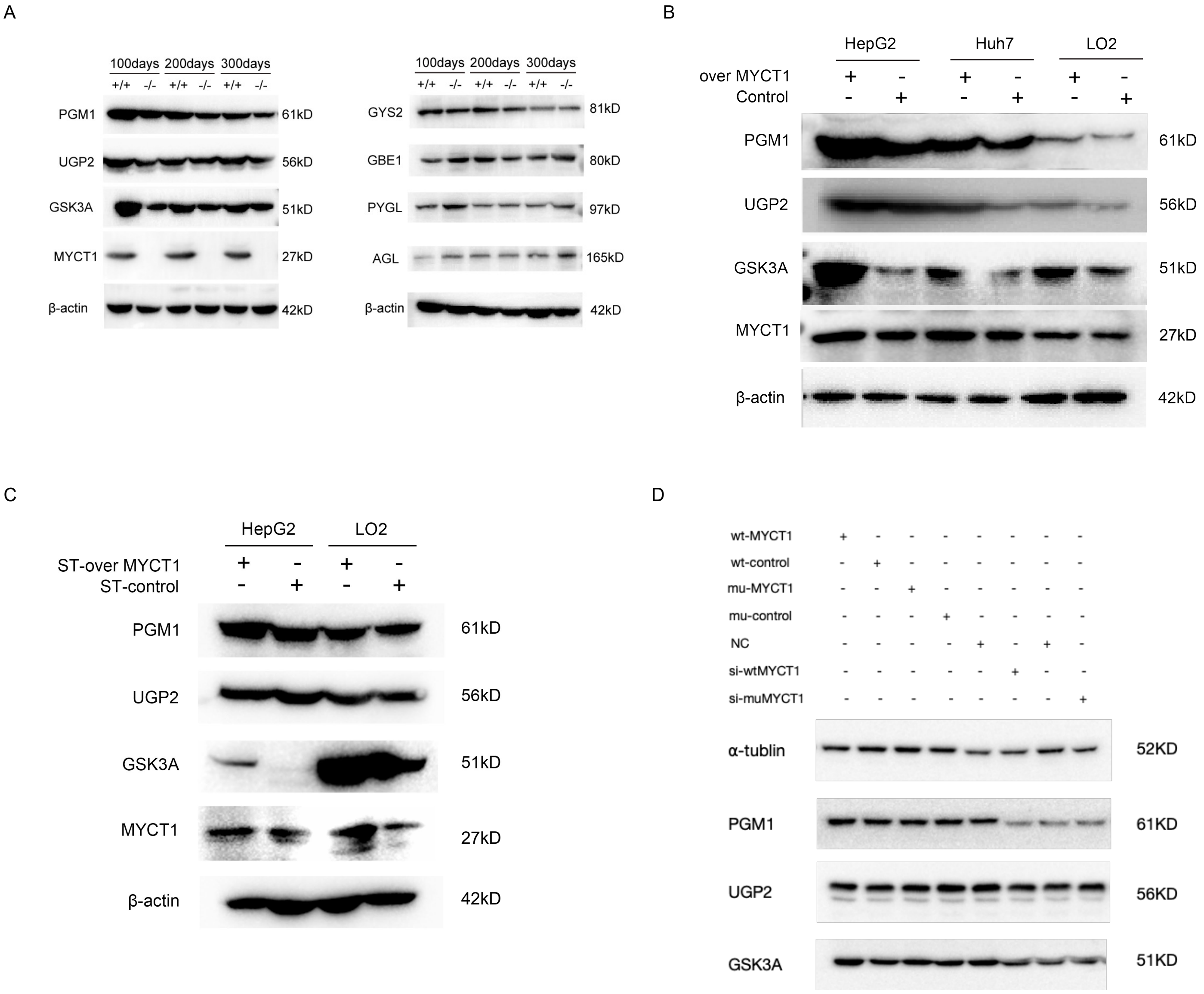


Figure S3. MYCT1 regulates the protein expression of glycogen enzymes. Related to Figure 4.

(A) Western blotting of glycogen-metabolizing enzymes in liver tissues from WT and *Myct1* KO mice aged 100 to 300 days.

(B) Western blotting of the glycogen-metabolizing enzymes PGM1, UGP2 and GSK3A in overMYCT1/control HepG2, Huh7 and LO2 cells.

(C) Western blotting of the glycogen-metabolizing enzymes PGM1, UGP2 and GSK3A in lentivirus-MYCT1 transfected LO2 and HepG2 cells.

(D) Western blotting of the glycogen-metabolizing enzymes PGM1, UGP2 and GSK3A in over WT-MYCT1/control, over MU-MYCT1/control, siWT-MYCT1/NC and siMU-MYCT1/NC HepG2 cells.

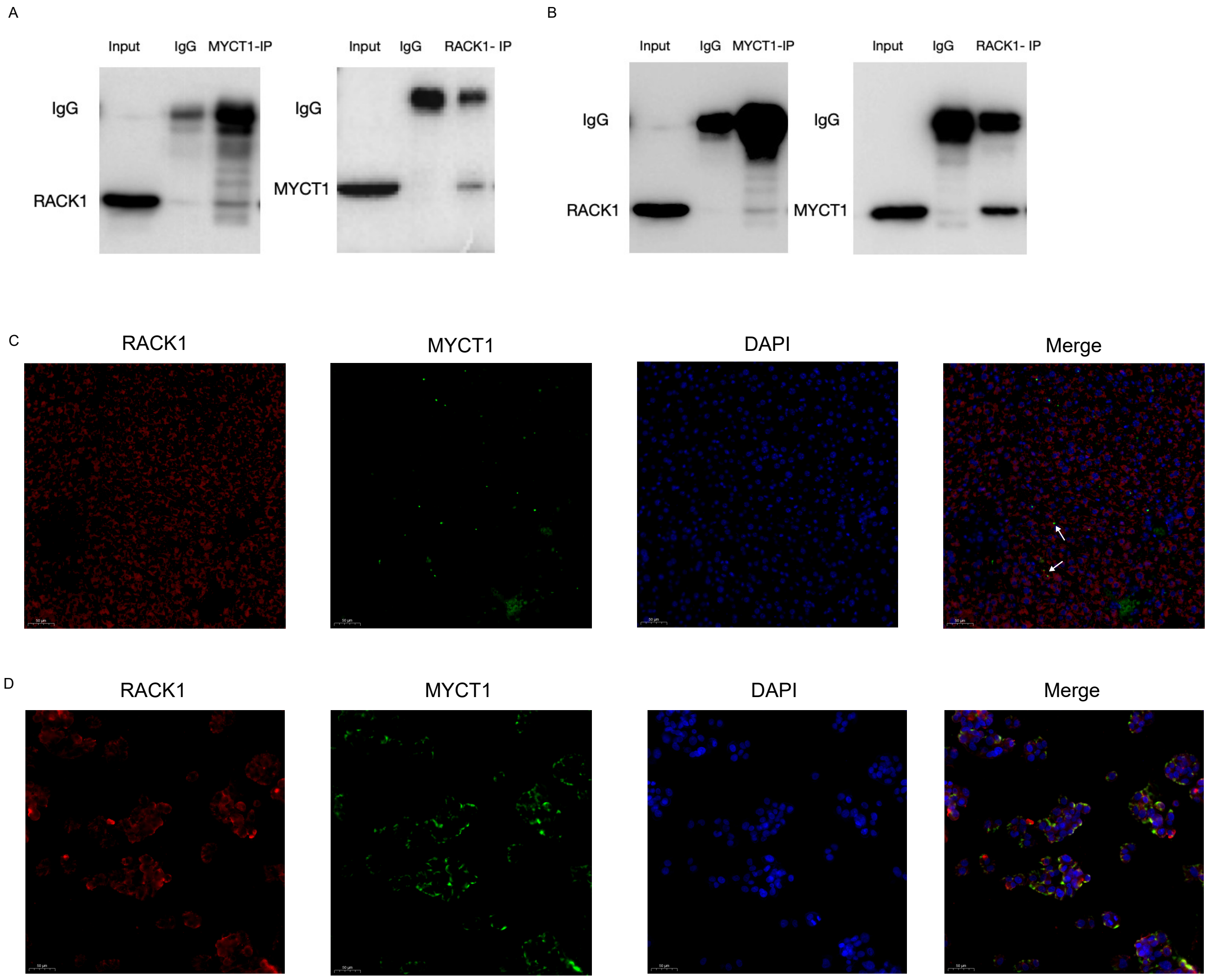


Figure S4. MYCT1 Interacts with RACK1. Related to Figure 5.

(A) Co-immunoprecipitation of MYCT1 and RACK1 in WT mice liver tissues.

(B) Co-immunoprecipitation of MYCT1 and RACK1 in HepG2 cells.

(C) Immunofluorescence stain of MYCT1 and RACK1 in lentivirus-overMYCT1/control HepG2 cells. Scale bar: 50 μm

(D) Immunofluorescence stain of MYCT1 and RACK1 in WT/*Myct1* KO mice liver tissues. Scale bar: 50 μm

**Table S1 The primer sequences for Real-time PCR. Related to STAR Methods**

| GENE | PRODUCT SIZE (bp) | PRIMER SEQUENCE |
|---|---|---|
| *CDS-Myct1* | 814 | F：5'-AGTATACGTGGGGTTGCGTG-3'<br>R：5'-TGTTCAGCTTTCACGAGCCT-3' |
| *Myct1* | 240 | F：5'-CCAAGGAAATCAAGCTTTAGGG-3'<br>R：5'-GGAATCGGGAAATGCCTTTATG-3' |
| *Pgm1* | 203 | F：5'-GCAGTTCTCAGCAAACGATAAA-3'<br>R：5'-CGTCCTTCTCGTAGCTATCAAT-3' |
| *Ugp2* | 96 | F：5'-GGTGCTTCTCAGTTCCAAGAGGTC-3'<br>R：5'-CTCATGTGAGGCTGCTGTGGTAAG-3' |
| *Gsk3a* | 116 | F：5'-GGCTGGCTGTGTACTTGCTGAG-3'<br>R：5'-TTTGTTCCCTGGTTGGCGTTCC-3' |
| *Actb* | 90 | F：5'-CTACCTCATGAAGATCCTGACC-3’<br>R：5'-CACAGCTTCTCTTTGATGTCAC-3' |
| *MYCT1* | 165 | F：5'-GCCAGAAAACTTTTGGGAGGA-3'<br>R：5'-ATCCAGTTCTGTTGAGGCCG-3' |
| *PGM1* | 101 | F：5'-CTTCAGCATTCCGTATTTCCAG-3'<br>R：5'-CAATCTTTGTAGCACTAGCCAC-3' |
| *UGP2* | 210 | F：5'-CCACAGCATCATCACATGAATT-3'<br>R：5'-CCACCACTAGTTTGTTCAACAC-3' |
| *GSK3A* | 131 | F：5'-AAAGGTGTTCAAATCTCGAACG-3'<br>R：5'-GACATCGCAGTTCATCAAAGAA-3' |
| *ACTB* | 90 | F：5’-GGGGTTCTCCCAACGTAAG-3’<br>R：5'-AACGCTTCACGAATTTGCGT-3' |
